# Public health round-up

**DOI:** 10.2471/BLT.16.010316

**Published:** 2016-03-01

**Medical supplies finally arrive in Taiz, Yemen**

A physiotherapist from the International Committee of the Red Cross examining a girl who lost part of her leg in an airstrike. Medical care and supplies are desperately needed in Yemen. After months of blocked access, the World Health Organization (WHO) finally succeeded in delivering more than 20 tonnes of essential medical supplies and medicines to the city of Taiz in Yemen, where more than 200 000 people are living under siege.

http://www.emro.who.int/media/news/who-medical-supplies-reach-taiz-city.html

**Figure:**
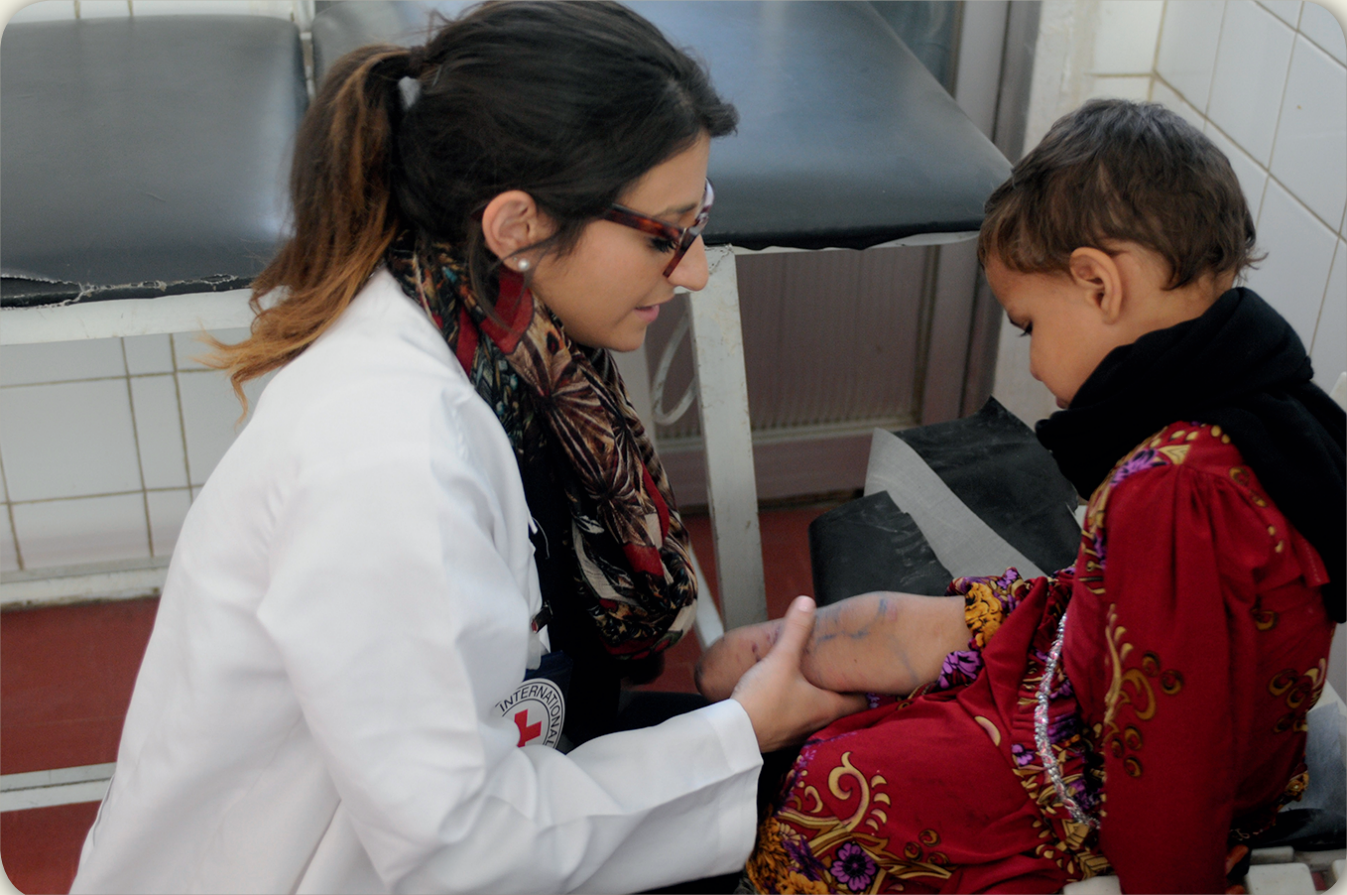
ICRC/Mohammed Abdullah

## Film ratings to fight tobacco 

Films with smoking scenes should be rated to prevent children and adolescents from seeing these scenes and starting to smoke cigarettes, according to a new WHO report, *Smoke-free movies: from evidence to action*. 

The report calls on governments to require health warnings to be shown before films containing smoking scenes, to ban the display of tobacco brands in films and to require film-makers to certify that they have not received payments to depict tobacco use. 

Citing growing evidence linking exposure to on-screen smoking with youth smoking initiation, the report argues that these measures can protect children from being introduced to tobacco products and subsequent tobacco-related addiction, illness and death. 

Since 2005, countries that are parties to the WHO Framework Convention on Tobacco Control have been required to adopt tobacco control measures, including a comprehensive ban on tobacco advertising, promotion and sponsorship. 

As a result, traditional outlets for tobacco advertising, such as magazines and television, are no longer available. However, the frequency of images showing smoking or other tobacco use in films has increased in many countries, the report notes. 

“With ever tighter restrictions on tobacco advertising, film remains one of the last channels exposing millions of adolescents to smoking imagery without restrictions,” said Dr Douglas Bettcher, Director of the Department of Prevention of Noncommunicable Diseases at WHO headquarters. 

“Smoking in films can be a strong form of promotion for tobacco products,” Bettcher said. “The 180 Parties to the WHO Framework Convention on Tobacco Control (WHO FCTC) are obliged by international law to ban tobacco advertising, promotion and sponsorship.” 

http://www.who.int/tobacco/publications/marketing/smoke-free-movies-third-edition

## Ending female genital mutilation

WHO highlighted the crucial role that health workers can play in helping to end the practice of female genital mutilation (FGM) on the international day of zero tolerance for female genital mutilation last month. 

Health professionals should be properly trained to recognize FGM in patients and provide better clinical care for women who have undergone some form of FGM, WHO said.

Health professionals should also be trained and empowered to resist families’ requests to perform FGM. 

FGM can be defined as any procedure that intentionally alters or causes injury to the female genital organs for non-medical reasons. It may have devastating physical, psychological, and social consequences for the women affected. 

“Health workers need evidence-based trainings to ethically manage requests by families to perform female genital mutilation, and accurately diagnose and manage women with complications from FGM,” said Dr Lale Say, a reproductive health expert at WHO. 

“Prevention and care require clinical guidelines and research to identify the optimal FGM management strategies,” she said. 

More research is needed on the care that can be offered to victims of FGM to prevent and manage its complications. 

Worldwide, more than 200 million girls and women have undergone FGM. Most live in 30 countries in the African, Eastern Mediterranean and South-east Asian regions.

http://www.who.int/reproductivehealth/topics/fgm/zero-tolerance-day

## Guinea-worm disease near eradication 

The number of cases of dracunculiasis– also known as Guinea-worm disease – decreased last year. 

Considerable progress has been made despite emerging challenges, but not all data from 2015 are available yet. The final results of the efforts to achieve the international goal of interrupting transmission by the end of 2015 will only be known later this year.

Guinea-worm disease is caused by the parasitic worm *Dracunculus medinensis*, which migrates through the victim's subcutaneous tissues causing severe pain and disability. 

The first symptom is intense itching, followed by a painful blister that usually appears on ankle or foot of the person infected. The worm eventually emerges from the blister. Its exit is normally accompanied by pain, fever, vomiting, nausea and diarrhoea. 

Only 22 cases were reported in 2015 in the four remaining endemic countries: Chad, Ethiopia, Mali and South Sudan. This is a reduction of 83% compared with 126 cases reported in 2014. 

“Security and access to endemic areas of Mali and South Sudan are the main challenges facing national eradication programmes,” said Dr Dieudonné Sankara from WHO’s Department of Control of Neglected Tropical Diseases. 

“Another challenge is the unusually high number of dogs with confirmed guinea-worm infections, particularly in Chad,” he said. 

Efforts to wipe out the disease globally started in the early 1980s. The first eradication goal was set for the end of 2009 and revised to the end of 2015. 

In 1989, Guinea-worm disease was endemic in 20 countries. To date, WHO has certified 198 countries, territories and areas as dracunculiasis-free. 

http://www.who.int/neglected_diseases/news/historic_low_number_GW_cases_reported

**Cover photo**

A woman looks out of her window in Brazil. A spike in the number of cases of microcephaly and Guillain-Barré syndrome has been observed in the Americas, these cases are suspected to be associated with Zika virus. Several Brazilian states are at the centre of the epidemic.

**Figure:**
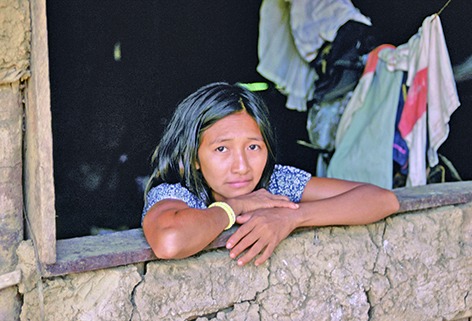
World Bank/Yosef Hadar

## Polio in Iraq

Iraqi children received protection from six major childhood diseases after the country’s introduction of the inactivated polio vaccine (IPV) as part of its national immunization programme. 

The IPV was introduced as a combination vaccine that contains antigens against polio, diphtheria, tetanus, pertussis, hepatitis B and *Haemophilus influenzae* type B. 

The introduction of IPV into routine immunization programmes is one of the key pillars in the global polio eradication effort. 

To date, the polio virus has been removed from every country in the world, apart from Afghanistan and Pakistan. 

In Iraq, the last laboratory-confirmed indigenous case of polio was reported in 2000. In 2014, two cases were reported, both linked to the 2013 outbreak in the Syrian Arab Republic.

In May 2015, Iraq was removed from the list of countries where polio had re-emerged following a series of vaccination campaigns supported by WHO, the United Nations Children’s Fund and other partners. 

http://www.emro.who.int/irq/iraq-news/inactivated-polio-vaccine-introduced-in-iraq.html

## Testing water treatment products

Eight out of 10 products evaluated in the first round of WHO’s International Scheme to Evaluate Household Water Treatment Technologies last year met WHO’s performance targets, according to results released this year. 

At least 1.9 billion people globally rely on water sources contaminated with faeces and could benefit from household treatment products to purify their water and, thus, protect their health. 

In 2014, WHO issued the first call to the manufacturers to submit expressions of interest for household water treatment products to be evaluated in the first round of the scheme. 

Ten products representing filtration, solar, ultraviolet and chemical disinfection methods were evaluated through laboratory testing and review of existing testing data. Two of them did not meet performance targets. 

“While WHO recognizes that performance is certainly not the only factor, there are no health benefits in distributing products that do not meet minimum health standards and doing so may mislead users,” stated the WHO report on the results of its Round I assessment.

Every year, WHO plans to test new technologies to help countries that are working to scale-up the use of effective household water treatment tools.

http://www.who.int/household_water/scheme/household-water-treatment-report-round-1

## Mental health meeting at World Bank

The World Bank and WHO will co-host an international meeting on global mental health next month to discuss how to scale up mental health services for depression and anxiety in primary care settings. 

The meeting entitled *Out of the shadows: making mental health a global priority* will coincide with the World Bank Group Spring Meetings in Washington, the United States of America, scheduled for 15 to 17 April. 

Mental health experts are due to present the case for investing more in cost-effective interventions for mental health. 

http://www.worldbank.org/en/topic/health/brief/mental-health

**Looking ahead**

**24 March – World Tuberculosis Day**.
**7 April – World Health Day**. The 2016 theme is diabetes.
**24–30 April – World Immunization Week**.
**23–28 May – Sixty-ninth World Health Assembly**, Geneva, Switzerland.
**23-24 May. World Humanitarian Summit**, Istanbul, Turkey. A global call to action by the United Nations Secretary-General Ban Ki-moon.
**14 June - World Blood Donor Day. **

